# Development of a Patient-Centered Preference Tool for Patients With Hematologic Malignancies: Protocol for a Mixed Methods Study

**DOI:** 10.2196/39586

**Published:** 2022-06-29

**Authors:** Amy Cole, Daniel R Richardson, Karthik Adapa, Amro Khasawneh, Norah Crossnohere, John F P Bridges, Lukasz Mazur

**Affiliations:** 1 Carolina Health Informatics Program University of North Carolina at Chapel Hill Chapel Hill, NC United States; 2 University of North Carolina Lineberger Comprehensive Cancer Center, University of North Carolina at Chapel Hill Chapel Hill, NC United States; 3 Division of Healthcare Engineering, Department of Radiation Oncology, School of Medicine, University of North Carolina at Chapel Hill Chapel Hill, NC United States; 4 Industrial Engineering Department, School of Engineering, Mercer University Macon, GA United States; 5 Department of Biomedical Informatics, The Ohio State University College of Medicine Columbus, OH United States

**Keywords:** co-design, informed decision making, mHealth, electronic health care tools, shared decision making, patient engagement, hematologic malignancies

## Abstract

**Background:**

The approval of novel therapies for patients diagnosed with hematologic malignancies have improved survival outcomes but increased the challenge of aligning chemotherapy choices with patient preferences. We previously developed paper versions of a discrete choice experiment (DCE) and a best-worst scaling (BWS) instrument to quantify the treatment outcome preferences of patients with hematologic malignancies to inform shared decision making.

**Objective:**

We aim to develop an electronic health care tool (EHT) to guide clinical decision making that uses either a BWS or DCE instrument to capture patient preferences. The primary objective of this study is to use both qualitative and quantitative methods to evaluate the perceived usability, cognitive workload (CWL), and performance of electronic prototypes that include the DCE and BWS instrument.

**Methods:**

This mixed methods study includes iterative co-design methods that will involve healthy volunteers, patient-caregiver pairs, and health care workers to evaluate the perceived usability, CWL, and performance of tasks within distinct prototypes. Think-aloud sessions and semistructured interviews will be conducted to collect qualitative data to develop an affinity diagram for thematic analysis. Validated assessments (Post-Study System Usability Questionnaire [PSSUQ] and the National Aeronautical and Space Administration’s Task Load Index [NASA-TLX]) will be used to evaluate the usability and CWL required to complete tasks within the prototypes. Performance assessments of the DCE and BWS will include the evaluation of tasks using the Single Easy Questionnaire (SEQ), time to complete using the prototype, and the number of errors. Additional qualitative assessments will be conducted to gather participants’ feedback on visualizations used in the Personalized Treatment Preferences Dashboard that provides a representation of user results after completing the choice tasks within the prototype.

**Results:**

Ethical approval was obtained in June 2021 from the Institutional Review Board of the University of North Carolina at Chapel Hill. The DCE and BWS instruments were developed and incorporated into the PRIME (*P*reference *R*eporting to *I*mprove *M*anagement and *E*xperience) prototype in early 2021 and prototypes were completed by June 2021. Heuristic evaluations were conducted in phase 1 and completed by July 2021. Recruitment of healthy volunteers began in August 2021 and concluded in September 2021. In December 2021, our findings from phase 2 were accepted for publication. Phase 3 recruitment began in January 2022 and is expected to conclude in September 2022. The data analysis from phase 3 is expected to be completed by November 2022.

**Conclusions:**

Our findings will help differentiate the usability, CWL, and performance of the DCE and BWS within the prototypes. These findings will contribute to the optimization of the prototypes, leading to the development of an EHT that helps facilitate shared decision making. This evaluation will inform the development of EHTs to be used clinically with patients and health care workers.

**International Registered Report Identifier (IRRID):**

DERR1-10.2196/39586

## Introduction

### Background

Hematologic malignancies include both indolent and highly aggressive blood cancers such as leukemia, lymphoma, and multiple myeloma. Outcomes for patients are heterogeneous based on disease and functional factors. The last decade has brought an unprecedented increase of Food and Drug Administration (FDA)–approved chemotherapeutic agents for patients with hematologic malignancies [1-6]. These approvals have expanded treatment options yet increased the complexity of treatment decisions. One primary role of clinicians is to elicit patients’ preferences to personalize treatment recommendations [7]. The standard shared decision-making process, however, is currently unreliable to accurately elicit patient preferences, frequently resulting in patient-provider preference-discordance [8-15]. Developing strategies to improve shared decision making is a key priority in advancing patient-centered care in oncology [16].

Novel stated preference methods including best-worst scaling (BWS) instruments and discrete choice experiments (DCEs) have recently been developed that can accurately quantify patient preferences [17,18]. BWS instruments have been used to prioritize the values of stakeholders [19]. Object case BWS instruments (henceforth simply referred to as BWS instruments) are thought to have less cognitive burden on respondents compared with other preference elicitation instruments [20]. Profile case DCEs (henceforth simply referred to as DCEs) are the most frequently employed type of DCE in health care. DCEs require participants to make choices between pairs of hypothetical treatments with different outcomes and have been particularly useful at rigorously quantifying the trade-off preferences of patients for treatments and informing patient-focused drug development [7]. Through a multistage process involving stakeholder engagement with patients, caregivers, and the FDA, we developed a BWS instrument and a DCE for patients with acute myeloid leukemia (AML) [19,21-23]. Using the BWS, we elicited the treatment preferences of 832 patients with AML and demonstrated that patients had the strongest concerns about outcomes in psychosocial and physical domains [19]. Using the DCE, we elicited specific treatment outcome preferences of 294 patients with AML and demonstrated substantial differences among preferences [22]. Some patients preferred to maximize their overall chance of long-term survival and were willing to endure a high burden of side effects or a lengthy hospitalization for this opportunity; others preferred to minimize treatment effects to maintain their quality of life.

DCEs and BWS instruments have been developed on paper and electronically [24]. To the best of our knowledge, comparative assessments of the usability, cognitive workload (CWL), and performance between DCEs and BWS instruments when developed as electronic health care tools (EHTs) have not been performed. This evaluation is important because use of EHTs has been shown to result in improved health outcomes, including increasing knowledge, improvement in risk perception, and improvement in communication between patients and health care workers [25]. In addition, involving older adults (>60 years of age) diagnosed with hematologic malignancies in the design process can positively impact their learning, sense of participation, and improve the development of the EHTs to better reflect the needs of the intended population [26].

### Prior Work

A paper version of the prototype was initially developed through a process involving multiple stakeholders including patients, advocacy groups, and researchers. This prototype was based on a DCE and developed specifically to elicit attribute preferences for treatment outcomes of patients with AML [22,23]. The DCE was piloted and then used in a national survey of patients with AML, who found it to be acceptable and feasible [27], and has been used prospectively in patients in an ongoing feasibility and acceptability trial with the UNC (University of North Carolina) Lineberger Comprehensive Cancer Center.

BWS is a simpler form of a DCE that does not involve changing levels of attributes. A BWS survey was developed by the principal investigator (DRR) of this study to capture the preferences of patients with AML and was used in a previous study, sampling 832 patients, illustrating that patients had the strongest concerns about treatment outcomes in psychosocial and physical domains [19,21].

Both the DCE and the BWS are algorithm based and were developed to quantify patient preferences based on a series of choice tasks, within which the number of attributes as well as the number of levels within each attribute determines the number of choice tasks required to assess preferences, as each attribute within each level has to be displayed a prespecified number of times to be validated [28].

### Objective

We aim to use mixed methods with an iterative co-design approach to develop an EHT that incorporates the concepts, tacit knowledge, and lived experiences of all stakeholders [29,30]. Use of a co-design process will not only contribute to the empowerment of stakeholders but also lead to the development of an EHT that helps patients and health care workers arrive at a therapy decision that is aligned with patient preferences. Our primary objective is to evaluate the perceived usability, CWL, and performance of the DCE and BWS within the prototypes.

## Methods

### Study Design and Methodology

#### Overview

**Figure 1:**
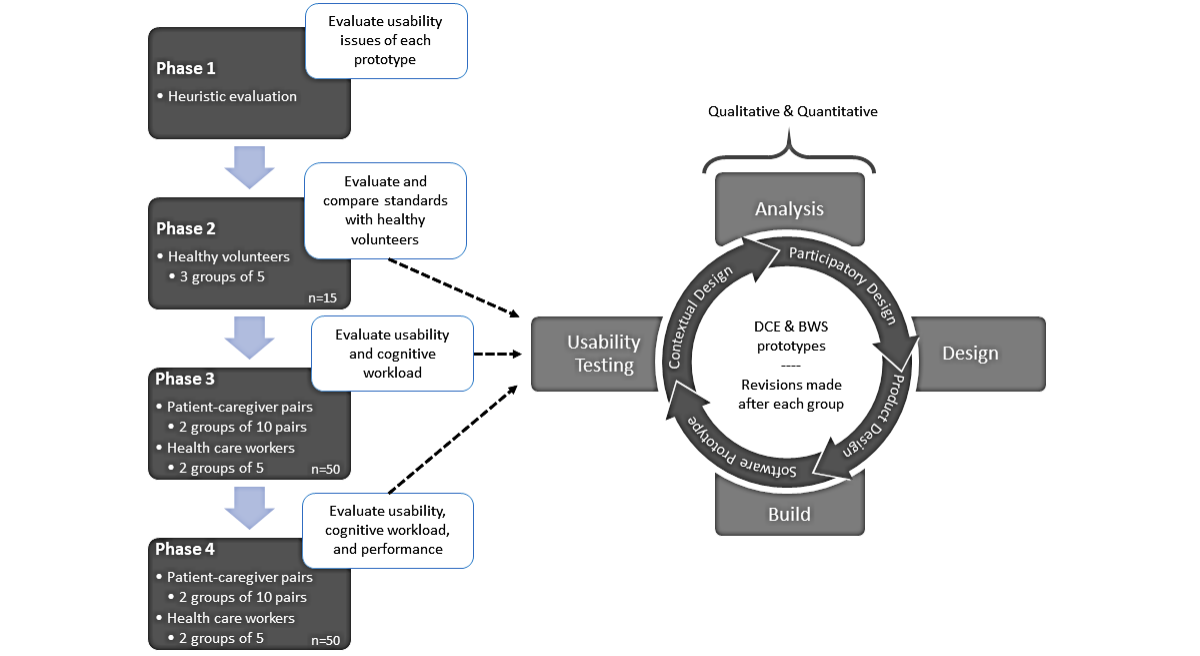
Study design workflow. BWS: best-worst scaling; DCE: discrete choice experiment.

We will use a mixed methods research approach utilizing an iterative co-design approach. In phase 1 we will first conduct heuristic evaluations on each prototype to identify and improve existing usability issues. Usability testing will then be conducted in 3 stages: (1) with healthy volunteers to establish standards for validating the results of the target population; (2) with patients and caregivers to obtain subjective and objective feedback based on both their tacit knowledge and their novel experience using each prototype; and (3) with health care workers using qualitative assessments to gain insight into their overall impression of the tool as well as whether they would recommend this to patients and caregivers. Within phases 2-4, participants will evaluate visualizations on a Personalized Treatment Preferences Dashboard, which shows a visualization of the results a user would see upon completion of the choice tasks within the prototype. We will evaluate the prototypes and visualizations using qualitative assessments (think-aloud sessions and semistructured interviews) to develop an affinity diagram for thematic analysis, and quantitative assessments using validated questionnaires to evaluate the perceived usability of and the CWL required to perform tasks within the prototypes. This study will be conducted in iterative development stages, in which feedback from participants will be analyzed and incorporated into the design that will then be presented to subsequent groups, as depicted in Figure 1.

The involvement processes utilized in this study will be classified by the participatory co-design framework originally developed by Leinonen [31] and further refined, representing 4 distinct groupings: contextual inquiry, participatory design, product design, and software prototype as hypothesis (functional prototype) [32], as depicted in Multimedia Appendix 1. Evidence gathered from this study will be used to develop a functional prototype that effectively elicits and displays preferences, leading to more informed treatment decisions.

#### DCE and BWS Choice Tasks

We used the established paper form of both the DCE and BWS to develop 2 medium-fidelity prototypes using Adobe XD version 40.0.22. All participants will access the prototypes through a study-provided iPad (8th generation). Each prototype is referred to as PRIME (*P*reference *R*eporting to *I*mprove *M*anagement and *E*xperience) but has distinct choice tasks as described below.

**Figure 2:**
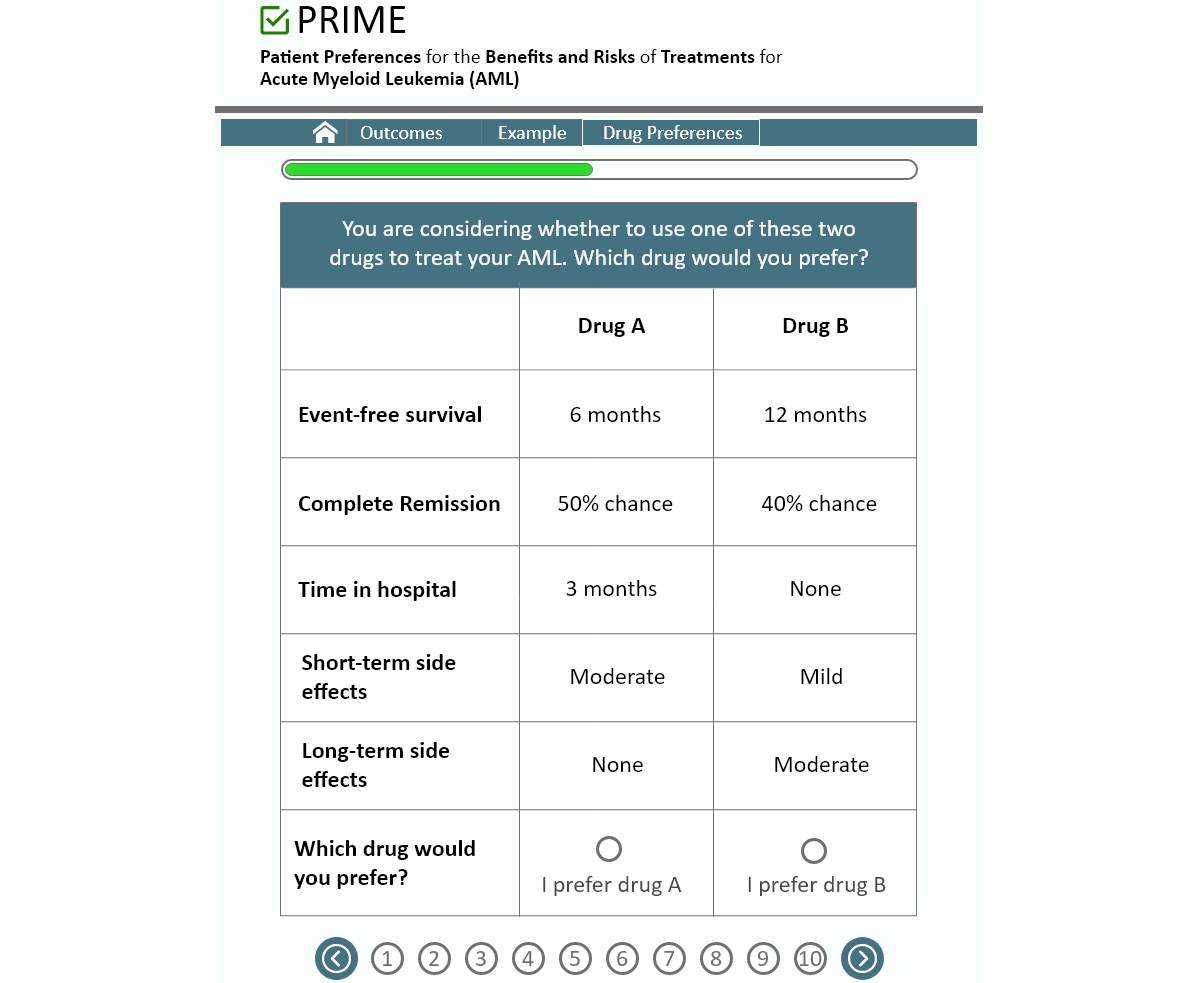
DCE choice tasks. DCE: discrete choice experiment; PRIME: *P*reference *R*eporting to *I*mprove *M*anagement and *E*xperience.

In the DCE, participants will go through a series of 10 hypothetical choice tasks, in which the same 5 attributes are presented (event-free survival, complete remission, time in hospital, short-term side effects, and long-term side effects) at different levels for each attribute within each task. For each choice task, participants will view differing profiles, consisting of attribute levels for “drug A” versus “drug B,” and will be asked to evaluate the benefits and risks for each, then select which drug they prefer, as depicted in Figure 2.

**Figure 3:**
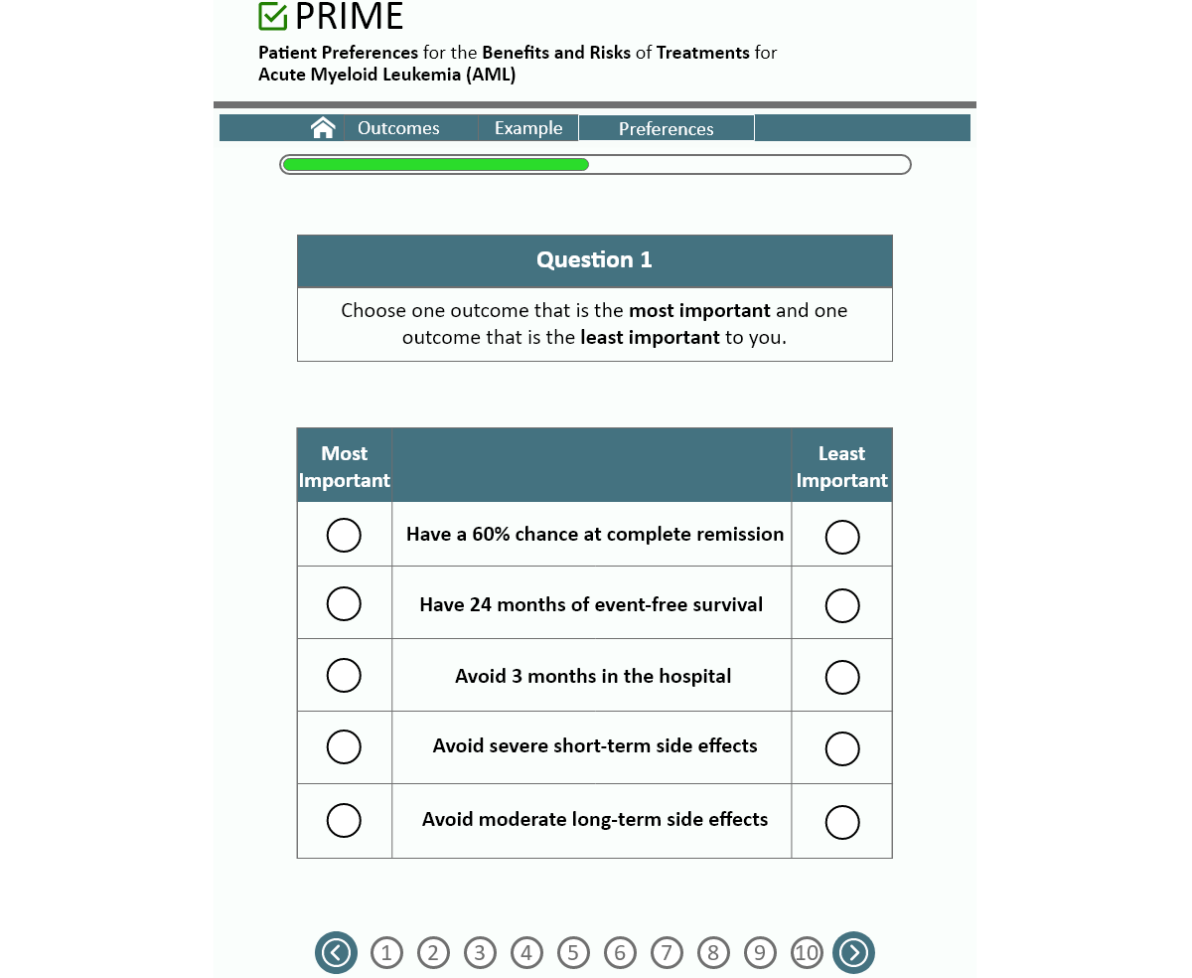
BWS choice tasks. BWS: best-worst scaling; PRIME: *P*reference *R*eporting to *I*mprove *M*anagement and *E*xperience.

In the BWS, participants will go through a series of 10 choice tasks, in which the same 5 attributes are presented (event-free survival, complete remission, time in hospital, short-term side effects, and long-term side effects), but the levels of each attribute vary within each task. For each choice task, participants will view 5 outcomes and will be asked to choose 1 outcome that is the most important and 1 outcome that is the least important to them, as depicted in Figure 3.

To ensure the same number of choice tasks are displayed to users when measuring performance (time to complete) between the DCE and BWS, a tenth task was added to the BWS. The tenth task will repeat choice task 2 and will not be used in quantifying patient preferences. However, as choice task 2 and choice task 10 are identical, this will provide evidence regarding the consistency with which respondents select answers within the prototype.

#### Visualization: Personalized Treatment Preferences Dashboard

Data from both the DCE and BWS prototypes will be used to generate a graphical representation of results based on the patients’ selected preferences. There is currently no standard of practice for generating visualizations of patient preferences. Therefore, we will elicit feedback on multiple visualizations in this study. Visualizations will be static representations and will not reflect the specific preferences of individual participants. To ensure the information presented is comprehensive and understandable, we have separated this from each prototype to solicit feedback specific to the visualization. Semistructured interviews will be conducted to obtain feedback on each visualization to improve participant understanding.

**Figure 4:**
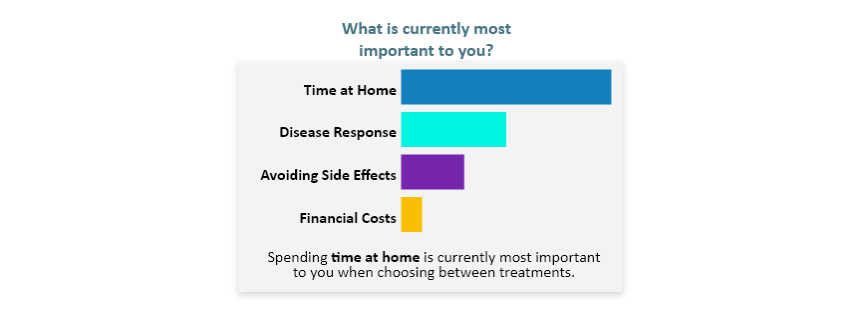
Bar chart.

**Figure 5:**
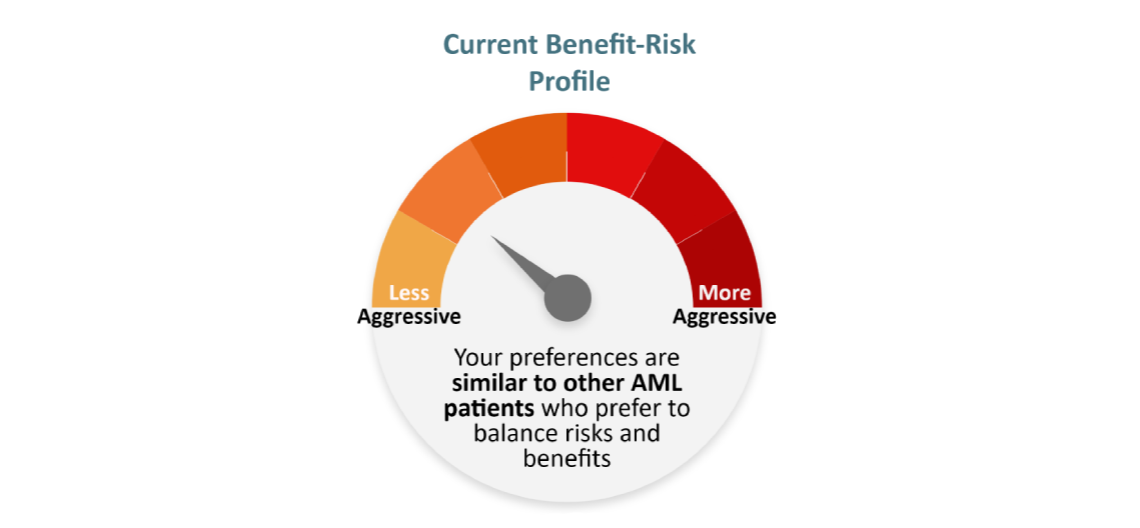
Gauge chart. AML: acute myeloid leukemia.

The first visualization (Figure 4) is a color-coded bar chart that displays the patients’ values in increasing levels of importance. The second visualization (Figure 5) uses a gauge to display the benefit-risk profile from a range of “less aggressive” to “more aggressive” with an arrow pointing to the patient’s specific level of aggressiveness in comparison to similar patients.

**Figure 6:**
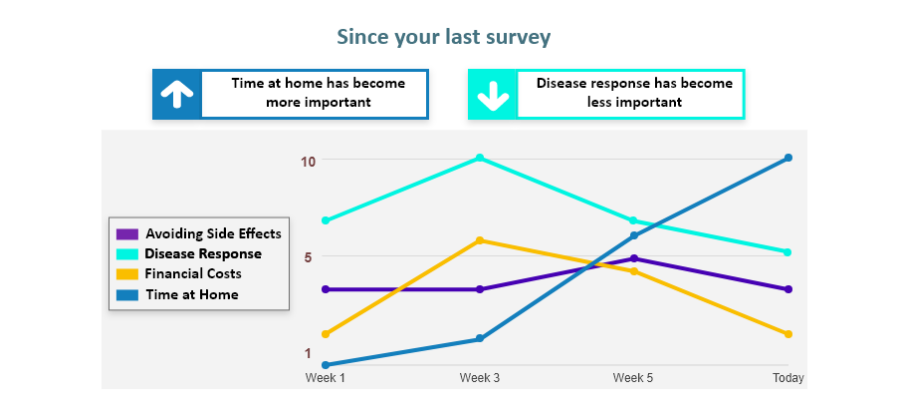
Preferences over time line graph.

The third visualization (Figure 6) is a line graph that displays how the patient’s preferences have changed over time.

**Figure 7:**
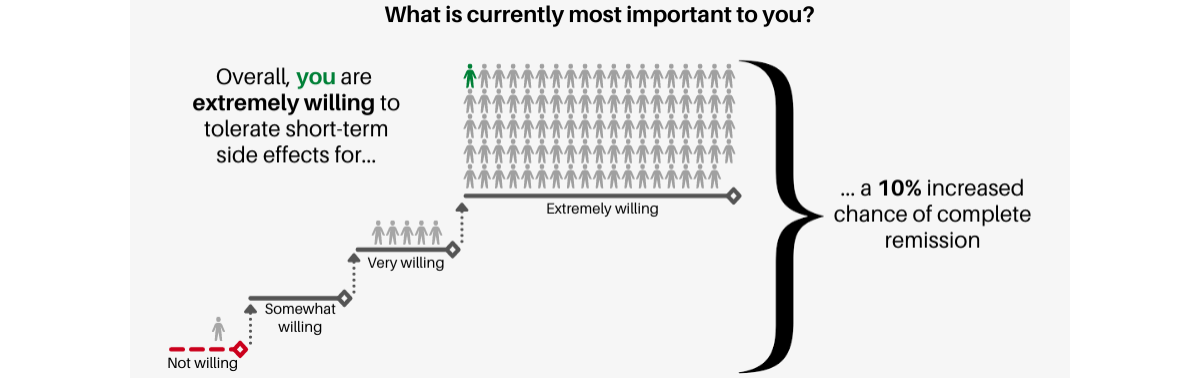
Narrative visualization.

The fourth visualization is a narrative visualization utilizing anthropomorphic icons to represent the patient relative to the population, as depicted in Figure 7. This visualization was informed by Segel and Heer’s [33] Narrative Visualization Framework and Munzner’s [34] Nested Model for Visualization Design. Specifically, Segel and Heer’s organization structure, consisting of 3 divisions, namely, (1) genre, (2) visual narrative tactics, and (3) narrative structure tactics, was utilized to gain a broader perspective on best techniques for visualizing preferences. Prior studies have demonstrated that risk recall is significantly higher using narrative visualization compared with other icon types when used in risk perception visualizations [35].

### Participants and Setting

This study involves 4 participant types including healthy volunteers, patients, caregivers, and health care workers. Healthy volunteers will be recruited from the general population through the Research for Me at UNC platform and must be 21 years or older [36]. Patients who meet the following criteria will be eligible to participate: (1) have a confirmed diagnosis of a hematologic malignancy including leukemia, lymphoma, or myeloma; (2) be on active chemotherapy (not including maintenance therapy for myeloma); (3) be able to read and understand English; (4) age 60 years or older; and (5) be willing to provide consent. Caregivers must be (1) 21 years and older, and (2) identified as the primary caregiver by the patient. Health care workers must be employed within hematologic malignancies (inpatient or outpatient) units as a nurse, nurse navigator, advanced practitioner, or physician. Administrators that oversee patients with hematologic malignancy and have experience working with patients with AML will also be eligible. Exclusion from the study will occur if participants have dementia, altered mental status, or a psychiatric condition that would prohibit the understanding or rendering of informed consent or participation in the user testing.

We have chosen to include patients, caregivers, and health care workers in the design process to better understand patients’ physical characteristics, the mental characteristics of patients and caregivers, the needs and behaviors of all participants, as well as the sociotechnical context in which each participant engages with technology and manages treatment decisions [37]. The purpose of this EHT is to facilitate dialog among patients, caregivers, and health care workers, and therefore it is important to understand the perspective of each stakeholder, and identify related themes and insights that will be used to define the direction of the final prototype [37]. We have chosen to include healthy volunteers to both establish standards for validating our results as well as to identify usability issues before conducting testing with our target population. We intend to engage all stakeholders in the design process by inviting them to participate at levels that will promote empowerment in the shared decision-making process [38].

Participants will be informed through the consent process that they will receive compensation for their participation in the study. Participants will receive a US $25 gift card upon completion of all tasks related to each PRIME prototype.

This study is taking place in the Human Factors Laboratory housed within the Department of Radiation Oncology at the University of North Carolina, Chapel Hill, NC. Eligible health care workers will participate in the study via an online session in Zoom (Zoom Video Communications, Inc.).

### Usability Testing and Data Collection

#### Phases and Measures

While some evaluation measures are conducted in all phases, as noted in Table 1, within each phase our study team will focus on a specific aspect of the prototype and data collection measure as described below.

**Table 1:** Usability testing objectives and measures.

Phase	Participants	Objective	Evaluation measures
Phase 1: Planning (heuristic evaluation)	Study team	To evaluate the general usability issues of each prototype	Heuristic evaluation
Phase 2: Evaluation with healthy volunteers	Healthy volunteers	To evaluate the usability and CWL^a^ of the DCE^b^ and BWS^c^ within the prototypes To evaluate the usability of the Personalized Treatment Preferences Dashboard	**Usability** Quantitative assessment: PSSUQ^d^ Qualitative assessments: Think-aloud sessions Semistructured interviews **Cognitive workload** Quantitative assessments: NASA-TLX^e^
Phase 3: Evaluation with patients, caregivers, and health care workers	Patients Caregivers	To evaluate the usability and CWL of the DCE and BWS within the prototypes	**Usability** Quantitative assessment: PSSUQ Qualitative assessments: Think-aloud sessions Semistructured interviews **Cognitive workload** Quantitative assessments: NASA-TLX Qualitative assessment: Cognitive task analysis
	Health care workers	To evaluate the usability of the DCE and BWS within the prototypes	**Usability** Qualitative assessments: Think-aloud sessions Semistructured interviews
Phase 4: Final evaluation of usability, CWL, and performance	Patients Caregivers	To evaluate the usability, CWL, and performance of the DCE and BWS within the prototypes	**Usability** Quantitative assessments: SEQ^f^ PSSUQ Qualitative assessments: Semistructured interviews **Cognitive workload** Quantitative assessments: NASA-TLX Qualitative assessment Cognitive task analysis **Performance** Quantitative assessments: Time to complete Number of errors Eye-tracking
	Health care workers	To evaluate the usability of the DCE and BWS within the prototypes	**Usability** Qualitative assessments: Think-aloud sessions Semistructured interviews

^a^CWL: cognitive workload.

^b^DCE: discrete choice experiment.

^c^BWS: best-worst scaling.

^d^PSSUQ: Post-Study System Usability Questionnaire.

^e^NASA-TLX: National Aeronautical and Space Administration’s Task Load Index.

^f^SEQ: Single Easy Questionnaire.

#### Phase 1: Planning (Heuristic Evaluation)

A group of 3-5 experts will conduct a heuristic evaluation of each prototype and provide feedback based on Dowding’s Usability Principles [39]. These principles incorporate Nielsen’s rating system [39,40] from “1=cosmetic problem only” to “4=usability catastrophe,” and consist of 7 general principles and 3 principles specific to information visualization. This process will ensure general usability issues are addressed. We anticipate that aggregating the results of our experts will help us to discover approximately 50%-75% of usability issues through this process, based on Nielson and Landauer’s model [41,42] for predicting usability problems. Dowding’s checklist of usability heuristics is an appropriate tool, as it provides guidance for evaluating both EHTs and data visualizations.

#### Phase 2: Evaluation With Healthy Volunteers

##### Overview

Healthy volunteers will be asked to complete a baseline questionnaire online via REDCap before the user testing session. Healthy volunteers will complete a short demographic questionnaire and will be asked to report on their race, ethnicity, education, household income, employment, and technology comfort level.

During the user testing session, healthy volunteers will be asked to compare and evaluate both the DCE and BWS prototypes as well as the visualizations within the Personalized Treatment Preferences Dashboard. Participants within this phase will be divided into 3 groups, each consisting of 5 users. Based on Nielson’s [43] user testing recommendations, user groups of 5 should be sufficient to resolve approximately 75% of usability issues.

Data gathered will be used to make iterative improvements to each prototype and then presented to subsequent groups for further evaluation.

Healthy volunteers will provide subjective and objective feedback about each prototype utilizing the following assessment methods.

##### Quantitative Assessments: Usability

Participants will evaluate the usability of each prototype by completing questions related to perceived usability in the Post-Study System Usability Questionnaire (PSSUQ). The PSSUQ is one of the most widely used poststudy standardized questionnaires, consisting of 16 questions divided into 3 subconstructs: system usefulness, information quality, and interface quality [44]. Based on 21 studies and 210 participants, the benchmark scores derived from Sauro and Lewis [45] provide the following means to interpret PSSUQ scores: overall (2.82), system quality (2.80), information quality (3.02), and interface quality (2.49). Better performance and satisfaction are reflected in a lower PSSUQ score [45]. The PSSUQ was chosen as it is a relatively quick assessment that can quantify the overall perceived usability of each prototype.

##### Quantitative Assessments: Cognitive Workload

The impact of each prototype on CWL will be quantified subjectively using the National Aeronautical and Space Administration’s Task Load Index (NASA-TLX) questionnaire. The NASA-TLX is widely considered to be a valid and reliable subjective measure of mental workload and is used across many disciplines [46-48]. The NASA-TLX measures 6 dimensions of CWL: mental, physical, and temporal demands, frustration, effort, and performance, with scores of 55 or more associated with reduced performance in numerous settings, including oncology [49]. NASA-TLX is considered to be the most commonly used subjective measure of CWL in health information technology and is a reliable measure of CWL in older adults [50]. As the NASA-TLX is easy to administer and has a low responder burden, this assessment was chosen as the appropriate measure to quantify the subjective CWL of users performing tasks within each prototype.

##### Qualitative Assessments: Usability

Participants will perform think-aloud sessions throughout the user testing to share how each prototype has been able to capture their preferences for treatment outcomes or whether the display of preferences is useful. We will engage participants as co-designers, soliciting feedback on the available functions in the current prototypes and encouraging them to make suggested improvements to the design of the EHT as well as the visualization depicting user preferences. Information obtained during user testing will be aggregated per group and used for iterative improvements to the prototype. Each group will evaluate prototypes that have been revised based on stakeholder feedback from the previous groups.

##### Qualitative Assessments: Semistructured Interviews

Semistructured interviews will be conducted to elicit feedback from participants on whether they understood various aspects of the tool, including but not limited to (1) definitions of the attributes, (2) ability to distinguish between the levels of attributes as presented, (3) which choice task series (BWS or DCE) was preferrable, and (4) whether they feel patients and family caregivers would utilize this tool. Data will be collected through the recording of interviews.

##### Participant Characteristics: Additional Validated Measures

Additional validated assessment measures will be included in the study to assess whether various attributes are correlated with treatment decisions (Multimedia Appendix 2). Participants will be assessed on memory skills, as well as on their electronic health literacy skills.

##### Participant Characteristics: Memory Skills

Participants will be asked to repeat a series of numbers during both the forward and backward assessments of the Digit Span test, a subset of the Wechsler Intelligence Scale, which when used separately, are considered validated measures of working memory [51,52]. Participants will be assessed on their ability to repeat strings of numbers, increasing in length by 1, until an error occurs on the reiteration of the string, or they reach a string length equal to 9. Participants will run through all forward strings before moving to the digit span backward test, in which they will be asked to repeat strings of numbers in reverse order. While the digit span test can be conducted by a verbal facilitator using only pencil and paper, we have chosen to use the computerized version, as there are benefits to the accessibility of both participants with hearing abilities and participants with hearing impairment, and elimination of verbal discrepancies (ie, rate or clarity of speech) that may exist among facilitators [53]. The computerized version of the digit span test is an appropriate measure to assess the working memory of users performing tasks on each prototype.

##### Participant Characteristics: Health Literacy

The eHealth Literacy Scale (eHEALS) and the eHealth Literacy Scale for Carers (eHEALS-Carer) are validated measures to assess the skills in acquiring health information through the use of technology, for oneself or the care of others, respectively [54]. The eHEALS assessment was developed to measure 6 skills: traditional literacy, health literacy, information literacy, scientific literacy, media literacy, and computer literacy [55]. The eHEALS model was further adapted to validate these same literacies among primary caregivers [56]. Each assessment consists of 8 questions, each measured on a 5-point Likert scale, ranging from “strongly agree” to “strongly disagree.” A score upon completion can range from 8 to 40, with higher scores representing higher eHealth literacy [57]. The eHEALS and eHEALS-Carer assessments are appropriate measures to evaluate the health literacy of users performing tasks on each prototype and will allow us to determine whether variability exists between participant types.

All in-person assessments and subjective measures have been converted to electronic surveys, and the data collected will be entered directly into REDCap. Paper assessments will be made available for participants who prefer this method, in which case all responses will then be manually transferred from the paper assessment to REDCap by a member of the study team.

#### Phase 3: Evaluation With Patients, Caregivers, and Health Care Workers

##### Overview

Participants will be asked to complete a baseline questionnaire online via REDCap before the user testing session. Questions related to the following domains will be asked of patients: race, ethnicity, education, household income, marital status, living situation, employment, technology comfort level, and insurance. Questions related to the following domains will be asked of caregivers: race, ethnicity, education, household income, marital status, living situation, employment, technology comfort level, insurance, length of time as a caregiver, and relationship to the patient. Health care workers will complete a short demographic questionnaire related to their role, their time in this role, and their exposure to patients with AML.

Further, patients will be asked to self-report on 2 of the 3 Simplified Geriatric Assessment (sGA) measures, which include the activities of daily living and the instrumental activities of daily living (Multimedia Appendix 2). The sGA tool was selected for reliability, brevity, and prognostic value in classifying the fitness level of older adults. Study coordinators will work with physicians to help participants complete the Cumulative Illness Rating Scale-Geriatric (CIRS-G), an assessment of comorbidity for enrolled patients [58]. Results from the activities of daily living, instrumental activities of daily living, CIRS-G, along with patient’s ages, will be calculated and used to classify patients as either fit, unfit, or frail [59].

If participants are unable to complete the self-reported preassessments online, they will be administered by study personnel either in person or over the phone.

Patient-caregiver pairs will be divided into 2 groups, each consisting of 10 patients and 10 caregivers. Patients and caregivers will be asked to participate in think-aloud sessions and semistructured interviews providing evidence on the usability of both the DCE and BWS within the prototypes, whether they understood various aspects of the tool, and whether they feel patients and family caregivers would utilize this tool. In addition to the assessments listed below, patients and caregivers will be involved in the same quantitative and qualitative assessments described in phase 2.

Health care workers will be divided into 2 groups, each consisting of up to 5 users. Health care workers will participate in qualitative assessments as described in phase 2 to assess the general usability of both the DCE and BWS within the prototypes and whether they would find this tool beneficial and would recommend its use for patients and caregivers.

Following recommendations from Francis et al [60], we have established sampling sizes within each group (up to 20 patient-caregiver pairs and 5 health care workers), a stopping criterion (set at 3 interviews), and the number of additional interviews we will conduct, to demonstrate that all themes have been introduced.

##### Qualitative Assessments: Cognitive Workload

Cognitive task analysis will be performed to gain insight into the thought processes and mental strategies of patients as they work through each prototype [61]. After completion of the task, a member of the study team will ask the participant to walk them through their process for selecting each treatment preference. Data will be collected through the recording of interviews.

##### Participant Characteristics

Patients and caregivers will complete the additional assessments (memory skills and health literacy) as described in phase 2. Patients will also take an additional assessment of cognition levels, as described below.

The Blessed Orientation Memory Concentration (BOMC) assessment is a validated measure of cognitive function. This assessment will be conducted through verbal facilitation by a study team member, in which patients will be asked to repeat and recall information, replicating their memory and concentration skills [62]. The final score is based on a weighted calculation and assesses the likelihood of cognitive disability, with a higher score representative of a clinically meaningful cognitive impairment [63]. The BOMC assessment tool is an appropriate measure to evaluate the memory and concentration of users performing tasks in each prototype.

#### Phase 4: Final Evaluation of Usability, CWL, and Performance

##### Overview

Patients, caregivers, and health care workers will complete the preassessments as described in phase 3. Patients and caregivers will continue to participate in quantitative and qualitative assessments as described in phase 3, excluding the think-aloud sessions. In phase 4, patients and caregivers will also be asked to participate in task analysis and performance assessments of the DCE and BWS within the prototypes, including the evaluation of tasks using the Single Easy Questionnaire (SEQ), tracking time to complete, and the number of errors.

Health care workers will continue to participate as described in phase 3.

##### Quantitative Assessments: Usability

The DCE and BWS choice tasks within each prototype will be treated as a task. During phase 3, participants will evaluate the usability of the prototypes by completing the SEQ after each task within each prototype. The SEQ is an experimentally validated tool and demonstrated as reliable, valid, and sensitive [64]. The SEQ is based on a 7-point scale, assessing how difficult users found a task, with average scores reported to be between 5.3 and 5.6 [64]. This posttask questionnaire will allow us to compare parts of the interface or workflows that are perceived as most problematic. As participants will be completing a series of assessments, we have chosen the SEQ as it is minimally disruptive, and helps understand participants’ attitudes toward the interface without subsequent tasks interfering with the user’s memory of the task just completed [47].

##### Quantitative Assessments: Performance

Participants will perform simulated tasks within the prototype and their responses will be recorded via the screen capture video software included in iPad OS14. As patients and caregivers in phase 4 perform tasks, they will be observed by 2 human factors experts and screen capture (touchscreen strokes, eye movement, and pupillary dilation into the captured video for analysis of pupillary response) will be performed. Their performance will be recorded as the time to complete each prototype, as well as the number of errors encountered.

##### Participant Characteristics

Patients and caregivers will complete the additional assessments as described in phase 3.

### Data Analysis

#### Statistical Testing

The study is primarily descriptive, as we aim to capture the usability, CWL, and performance of participants using EHTs that include the DCE and BWS instruments.

Within-subjects testing will be conducted to compare preferences regarding the presentation of information, usability, CWL, and performance of the DCE and BWS within the prototypes. The order of presentation will be randomized for each participant to account for the effect of order and to ensure that information is not transferred across prototypes [65].

#### Quantitative

The paired *t* test will be utilized to evaluate the statistical significance of the PSSUQ, SEQ, NASA-TLX, and performance (time taken for task completion and the number of errors committed) of participants using both the DCE and BWS within the prototypes for each group in all relevant phases. Analysis of variance tests will be utilized to evaluate whether statistical differences in the DCE and BWS occur across groups for these same measures. We will consider *P* values of less than .05 to have statistical significance, and 95% CIs will be used to establish differences between the DCE and BWS both within and across groups.

We aim to use the data from preassessments (demographics, geriatric assessment), in-person assessments (cognition, memory, and health literacy), digit span, and health records to determine predictor variables with patient treatment preferences, and to determine whether health literacy variance exists between patients and caregivers. Analysis of preassessments will include basic statistics (eg, mean, SD, and range).

All analysis will be performed in SAS JMP Pro (SAS Institute) for summarizing and grouping study results both between and across groups. We will work with an experienced statistician to ensure that we utilize appropriate methods and theories for data analysis.

#### Qualitative

Qualitative data obtained through think-aloud sessions, interviews, and cognitive task analysis will be analyzed for each group and coded using thematic analysis [66,67]. Analysis of qualitative responses will include creating contingency tables and converting data into charts and graphs for identifying patterns and gaps. One-way contingency tables will be used to evaluate whether participants (1) found the prototypes challenging to complete, (2) understood the definitions of the attributes, (3) could distinguish between the attribute levels, (4) preferred either the DCE or BWS, and (5) felt patients or family caregivers would use this tool.

### Ethics Approval

This study, reference ID number 321807, received institutional review board (IRB) approval from the University of North Carolina Office of Research Information Systems in June 2021. It has been determined that the risk involved in this research is no more than minimal. This research requires annual UNC administrative review. Under the revised “Common Rule” of 2018, this study does not require continuing review and IRB approval will not expire. This study was reviewed in accordance with federal regulations governing human subjects research, including those found at 45 CFR 46 (Common Rule), 45 CFR 164 (HIPAA [Health Insurance Portability and Accountability Act]), 21 CFR 50 & 56 (FDA), and 40 CFR 26 (EPA), where applicable.

### Privacy and Security

We will do a chart review on patient electronic medical records, including age, race, treatment, and diagnosis. A message will be sent to the treating physician (oncologist) or principal investigator (DRR) to inquire as to whether the patient would be appropriate for the study.

The interview and usability testing will be conducted in the human factors laboratory that can be accessed only by the study team. In the laboratory, participants and researchers can work from their respective workstations, which are separated by glass and can communicate effectively using the devices available in the laboratory.

Health care workers will complete user testing via Zoom, which is HIPAA enabled through the UNC School of Medicine. The other questionnaires (eg, geriatric assessments) will be sent to the participants using REDCap, a secure web application. During the data collection in the laboratory, we will ensure that those not connected with this study are unable to see participants or hear the information that is shared. When reporting the research findings, data will be presented in a way that prevents individual participants from being identified.

This trial will be audited by the Lineberger Comprehensive Cancer Center data safety and monitoring committee every 6 or 12 months.

## Results

This study received approval from the Lineberger Comprehensive Cancer Center Oncology Protocol Review Committee in March 2021 and IRB approval from the University of North Carolina Office of Research Information Systems in June 2021. We began recruitment of healthy volunteers in August 2021. This study is expected to conclude in February 2023.

## Discussion

### Principal Findings

To the best of our knowledge, this study is the first to conduct comparative assessments of the usability, CWL, and performance between DCEs and BWS instruments when developed as an EHT. We designed this as a mixed methods study using an iterative co-design approach that includes healthy volunteers, patients, caregivers, and health care workers. One limitation of this methodology is that participants in phase 2 of this study are all healthy volunteers and likely have different life experience to our target population of older adults with hematologic malignancies. While our target population will likely experience the EHT differently than these volunteers, our study team anticipated that the findings from both qualitative and quantitative assessments would lead to usability improvements for phase 3 participants, which include patients and caregivers. This is consistent with previous studies that have first recruited healthy volunteers to effectively resolve usability problems prior to the definitive work with the target population [68,69].

### Dissemination

In December 2021, our findings from phase 2 were accepted for a publication in the conference proceedings in Springer Nature and for presentation at the virtual Human-Computer Interaction 2022 Conference [70]. Our study team is highly interdisciplinary; therefore, we aim to disseminate our findings across a more diverse audience within both medicine and health care engineering conferences and journals.

### Conclusion

Our findings will help differentiate the usability, CWL, performance, and alignment of patient preferences for both the DCE and BWS within the prototypes. This study has the potential for optimizing the collaboration and empowerment of older adults in the development of an EHT, improving elicitation of treatment preferences, and improving communication between patients and health care workers. Future research should continue to evaluate the levels at which older adults participate in the full development process, and how this can either limit or encourage their collaboration and empowerment.

## Appendix

Multimedia Appendix 1 Proposed involvement processes based on Leinonen's framework.

Multimedia Appendix 2 Proposed validated assessments.

## Abbreviations

AML: acute myeloid leukemia
BOMC: Blessed Orientation Memory Concentration
BWS: best-worst scaling
CIRS-G: Cumulative Illness Rating Scale-Geriatric
CWL: cognitive workload
DCE: discrete choice experiment
eHEALS: eHealth Literacy Scale
eHEALS-Carer: eHealth Literacy Scale for Carers
EHT: electronic health care tool
FDA: Food and Drug Administration
HIPAA: Health Insurance Portability and Accountability Act
IRB: institutional review board
NASA-TLX: National Aeronautical and Space Administration’s Task Load Index
PRIME: *P*reference *R*eporting to *I*mprove *M*anagement and *E*xperience
PSSUQ: Post-Study System Usability Questionnaire
SEQ: Single Easy Questionnaire
sGA: Simplified Geriatric Assessment
UNC: University of North Carolina

## Data Availability

The data sets generated during or analyzed during this study are available from the corresponding author on reasonable request.
