# Supplementary material for: Development of a Patient-Centered Preference Tool for Patients With Hematologic Malignancies: Protocol for a Mixed Methods Study
Source: JMIR Res Protoc. 2022 Jun 29;11(6):e39586. doi: 10.2196/39586 (PMC9280452; doi:10.2196/39586)
Supplement: Multimedia Appendix 1 [file resprot_v11i6e39586_app1.pdf]

Development of a patient-centered preference tool for patients with hematologic malignancies: protocol for a mixed methods study

Multimedia Appendix 1

Proposed Involvement Processes based on Leinonen's framework

Involvement Processes	Study Team	Patient	Caregiver	Healthcare Worker	Healthy Volunteer
Contextual Inquiry[a]				X	
Survey (baseline participant questionnaire)[b]		X	X	X	X
Interviews [b]		X	X	X	X
Think aloud [b]		X	X	X	X
Remote evaluation [b]				X	
Task analysis [b,d]		X	X		X
Prototype – medium-fidelity/throw-away [c]		X	X	X	X
Heuristic evaluation [c]	X				

Contextual Inquiry[a]
Participatory Design[b]
Product Design[c]
SEQ, PSSUQ, NASA-TLX, Eye-Tracking, Performance (Time to Complete/Number of Errors)[d]
