# Supplementary material for: Development of a Patient-Centered Preference Tool for Patients With Hematologic Malignancies: Protocol for a Mixed Methods Study
Source: JMIR Res Protoc. 2022 Jun 29;11(6):e39586. doi: 10.2196/39586 (PMC9280452; doi:10.2196/39586)
Supplement: Multimedia Appendix 2 [file resprot_v11i6e39586_app2.pdf]

Development of a patient-centered preference tool for patients with hematologic malignancies: protocol for a mixed methods study

Multimedia Appendix 2

*Proposed Validated Assessments*

| Validated Assessments | Patient | Caregiver | Healthy Volunteer |
|---|---|---|---|
| Geriatric Assessment (self-reported)[a] | X | | |
| Digit Span | X | X | X |
| eHEALS | X | | X |
| eHEALS-Carer | | X | |
| Blessed Orientation Memory Concentration (BOMC) | X | | |

Activities of Daily Living (ADL) [a]

Instrumental Activities of Daily Living (IADL) [a]
